# Netrin-1 and Semaphorin 3A Predict the Development of Acute Kidney Injury in Liver Transplant Patients

**DOI:** 10.1371/journal.pone.0107898

**Published:** 2014-10-07

**Authors:** Lidia Lewandowska, Joanna Matuszkiewicz-Rowińska, Calpurnia Jayakumar, Urszula Oldakowska-Jedynak, Stephen Looney, Michalina Galas, Małgorzata Dutkiewicz, Marek Krawczyk, Ganesan Ramesh

**Affiliations:** 1 Vascular Biology Center, Georgia Regents University, Augusta, GA, United States of America; 2 Department of Nephrology, Dialysis & Internal Diseases, Medical University of Warsaw, Warsaw, Poland; 3 Department of General, Transplant and Liver Surgery, Medical University of Warsaw, Warsaw, Poland; 4 Department of Biostatistics and Epidemiology, Georgia Regents University, Augusta, GA, United States of America; 5 Department of General and Nutritional Biochemistry, Medical University of Warsaw, Warsaw, Poland; University of Torino, Italy

## Abstract

Acute kidney injury (AKI) is a serious complication after liver transplantation. Currently there are no validated biomarkers available for early diagnosis of AKI. The current study was carried out to determine the usefulness of the recently identified biomarkers netrin-1 and semaphorin 3A in predicting AKI in liver transplant patients. A total of 63 patients’ samples were collected and analyzed. AKI was detected at 48 hours after liver transplantation using serum creatinine as a marker. In contrast, urine netrin-1 (897.8±112.4 pg/mg creatinine), semaphorin 3A (847.9±93.3 pg/mg creatinine) and NGAL (2172.2±378.1 ng/mg creatinine) levels were increased significantly and peaked at 2 hours after liver transplantation but were no longer significantly elevated at 6 hours after transplantation. The predictive power of netrin-1, as demonstrated by the area under the receiver-operating characteristic curve for diagnosis of AKI at 2, 6, and 24 hours after liver transplantation was 0.66, 0.57 and 0.59, respectively. The area under the curve for diagnosis of AKI was 0.63 and 0.65 for semaphorin 3A and NGAL at 2 hr respectively. Combined analysis of two or more biomarkers for simultaneous occurrence in urine did not improve the AUC for the prediction of AKI whereas the AUC was improved significantly (0.732) only when at least 1 of the 3 biomarkers in urine was positive for predicting AKI. Adjusting for BMI, all three biomarkers at 2 hours remained independent predictors of AKI with an odds ratio of 1.003 (95% confidence interval: 1.000 to 1.006; *P* = 0.0364). These studies demonstrate that semaphorin 3A and netrin-1 can be useful early diagnostic biomarkers of AKI after liver transplantation.

## Introduction

Acute kidney injury (AKI) is a serious complication after liver transplantation. Several studies have demonstrated that development of AKI has been associated with increased length of hospital stay, morbidity, and mortality [1]–[3]. The incidence of post liver transplantation AKI has been reported in the range of 50–94% [2]–[5]. The mechanisms of renal dysfunction in liver transplant recipients are not clearly understood. Calcineurin inhibitors are generally perceived as the most prominent cause; however, the liver transplant procedure itself represents a significant surgical/hemodynamic/inflammatory trauma that, on its own can cause renal dysfunction [1]. Development of therapies has been hindered by lack of early diagnostic biomarkers. Creatinine and creatinine clearance are late markers of AKI, and changes in these parameters occur only after substantial injury has already occurred. Even a stable creatinine level does not exclude structural kidney damage. Moreover, in the settings of end stage liver disease, creatinine was found to be an unreliable marker of renal function [6]. Several studies have examined urinary neutrophil gelatinase-associated lipocalin (NGAL), IL-8, IL-18 and liver fatty acid binding protein (L-FABP) as biomarkers of AKI in orthotopic liver transplantation (OLT) patients [7], [8]. However, their usefulness has not been translated to theclinic. The recent identification of new biomarkers e.g., netrin-1 and semaphorin 3A (sema3A) for the diagnosis of AKI have shown significant promise in a variety of clinical settings [9]–[12]. However, the use of these biomarkers for the detection of AKI in patients undergoing OLT has not been explored. Netrin-1 and sema3A are neuronal guidance cues known to regulate axons to find their target during development [13]–[16]. However, these molecules are also expressed in adult tissues, including kidney. Recent studies show that netrin-1 plays a protective role in kidney injury, whereas sema3A may have a pathogenic role [17]–[19]. Therefore, in this study, we examined these newly identified biomarkers, netrin-1 and sema3A, in OLT patient urine to determine whether they can predict the early development of AKI, much before serum creatinine levels are increased.

## Materials and Methods

### Patients

All patients over 18 years old who underwent an OLT at the University of Warsaw Medical Center, Poland were eligible for the study. The age of the patient populationranged from 19 to 64 years. The inclusion criteria included liver insufficiency, acute or chronic, such that liver transplantation was required. Patients were excluded from the study if they were unable to provide consent and if they had had a previous transplant procedure. A total of 68 patients were recruited, out of which 4 patients were excluded due to a second transplant procedure and 1 patient was excluded due to incomplete collection of urine samples. I All donors over age 60 years and donors aged 50–59 years with at least two of the three following criteria (1) cause of death was cerebrovascular accident; 2) preexisting history of systemic hypertension; and 3) terminal serum creatinine >1.5 mg/d were identified as extended donor criteria (EDC)). Whether or not the organ donor was an EDC did not appear to affect whether the receipient did or did not develop AKI. Ten patients had preexisting renal dysfunction (mean basal serum creatinine concentration 2.14±1.19 mg/dl; range 1.26–5.31 mg/dl). All transplanted patients received either tacrolimus, cyclosporine, or mycophenolate mofetil as immunosuppressive therapy. All eligible patients were consented according to proper procedures over an 18-month period (2010–2011) were enrolled at the hospital. All study enrollment procedures and subsequent data collection and acquisition were approved by the Institutional Review Board at the Medical University of Warsaw. Written informed consent was obtained from the patients or their legal guardian if they were not capable of consenting before enrollment. The sample processing and data analysis were carried out at Georgia Regents University and were approved by Institutional Review Board.

### Sample collection and processing

Pre-operative blood and urine samples (ten milliliters) were collected from each patient within 24 hours of OLT. Additional urine samples (10 milliliter) were collected at 2, 6 and 24 hr after OLT (measured from the time the operation ended). Blood samples were collected at 2, 6 and 24 hr, then every 24 hr thereafter until day 7 after surgery. Collected urine samples were centrifuged at 10000 G for 10 minutes, and the supernatant liquid was aliquoted and stored at −80°C. Blood samples were centrifuged at 10000 RPM for ten minutes, and serum was stored at −80°C.

### Biomarker Measurement

Biomarkers were measured on individual, not pooled, samples. Urine netrin-1 levels were measured with a specific enzyme-linked immunosorbent assay (ELISA) kit (Cat #MBS725887, MyBiosource, Inc., San Diego, CA) that specifically detects human netrin-1. Urine sema3A levels were measured with a specific ELISA kit (Cat #MBS732622, MyBiosource, Inc., San Diego, CA). All measurements were made in a blinded fashion. The inter- and intra-assay coefficient variations were 5–10%, corresponding to that reported by the kit manufacturer. Urine NGAL levels were measured using a commercially available ELISA kit (Human Lipocalin-2/NGAL, R&D Systems, Minneapolis, MN, cat no. DLCN20).

Serum creatinine levels were measured daily by the hospital’s central laboratory as part of routine peri-operative care, but we performed additional measurements corresponding to same time-points used here for the biomarker measurements, and then additionally for 7 days of follow-up observation. A combination of retrospective and prospective chart reviews was performed to collect demographic and pertinent clinical data. AKI was defined as an increase in serum creatinine level by 50% or greater compared to pre-operative values that occurred within 72 hours of the OLT. Results were also analysed according to the definition of AKI, which was based on the Risk, Injury, Failure, Loss, End-Stage Renal Disease (RIFLE) classification [20], [21].

### Statistical Analyses

SAS version 9.3 was used for all analyses (SAS Institute, Cary, NC, 2010), and a significance level of 0.05 was used throughout, controlling for multiple comparisons whereever necessary. Demographics and clinical outcomes were compared between patients who developed AKI and patients who did not. Continuous variables were compared using the two-sample *t* test, and categorical variables were compared using Fisher’s exact test. Estimates of mean values of serum creatinine and urinary netrin levels by a group at various time points were calculated using repeated-measures ANOVA, which accounts for correlations of measurements from the same individuals across time. Least square (LS) means and their standard errors (SEMs) are reported. Spearman correlation coefficients were used to examine the correlation between urinary netrin concentrations at various time points (baseline and at 2, 6, and 24 hours after surgery) and the following clinical outcomes: percent change in serum creatinine level, liver transplantation surgery time, length of hospital stay after surgery and days of AKI.

To measure the sensitivity and specificity for urinary netrin-1, a conventional receiver-operating characteristic (ROC) curve was generated for urinary netrin at 2, 6, and 24 hours after liver transplantation. We calculated the area under the curve (AUC) to ascertain the utility of netrin-1 as a biomarker. An area of 0.5 is expected by chance, whereas a value of 1.0 signifies a perfect biomarker. The optimal urinary netrin time point was selected to maximize prediction at the earliest time possible, thus weighing the AUC, timing of measurement, and *P* value from the predictive logistic model. We then identified the values of urinary netrin level that provided 95% sensitivity, 95% specificity, and optimal sensitivity and specificity using the ROC curve at the best time point.

Univariable and multivariable logistic regression analyses were then performed to assess predictors of AKI. Potential independent predictor variables included urinary netrin concentration at the best time point, age, sex, BMI, surgery time, hospital length of stay and urine output on day 1. Variables were retained in the final model if *P*≤0.05.

## Results

### Patient Characteristics and Renal Function Changes

**Figure 1:**
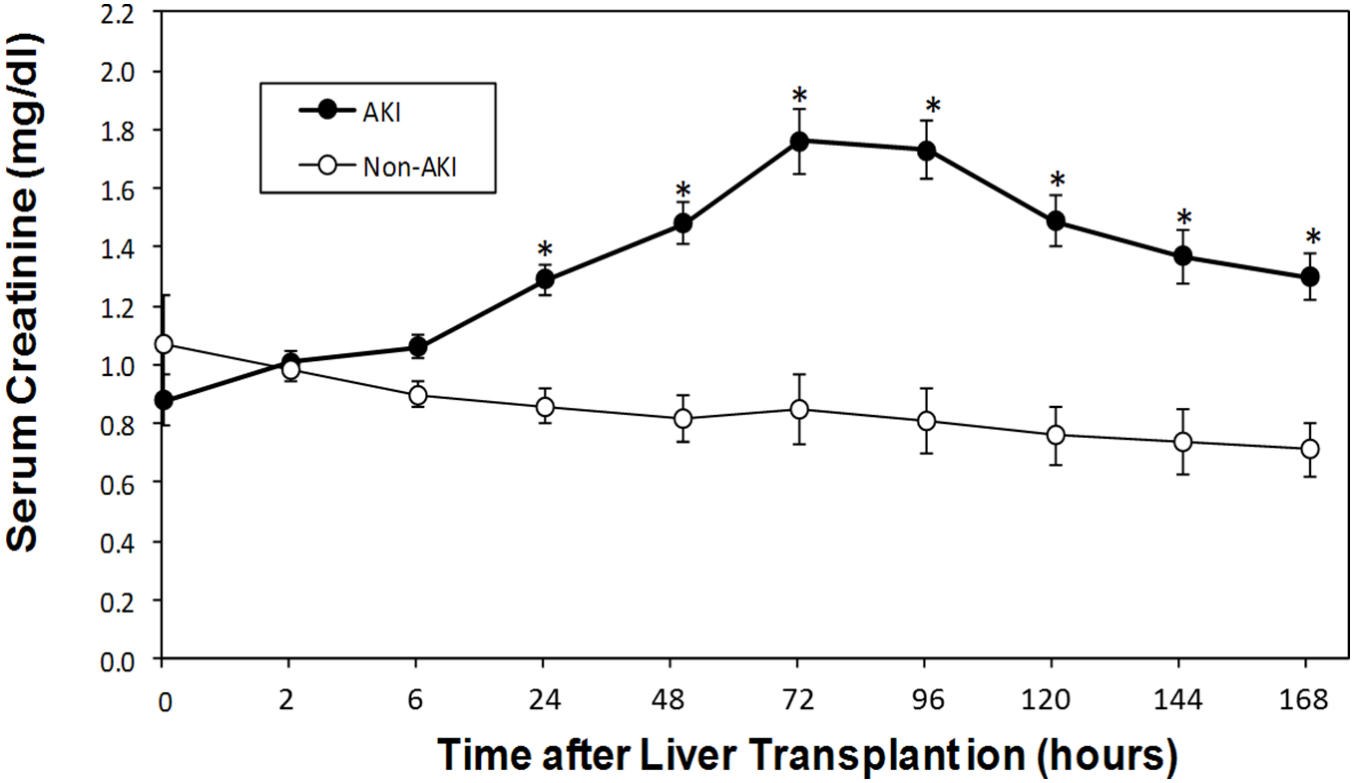
Changes in serum creatinine (LS mean ± SE) at various time points after liver transplantation in the non-AKI and AKI group. *p≤0.0001 for differences between groups by repeated measures ANOVA.

During the enrollment period, 83 subjects underwent a liver transplant at our institution. Of these, 63 subjects met the inclusion criteria for this study. AKI occurred in 35 patients (56%) within a 3-day period. No significant differences were noted between the two groups with respect to gender, BMI, surgery time, or hospital stay (Table 1). Patients who developed AKI were significantly older at baseline compared with those who did not (*P* = 0.0475) and had significantly greater percent change in serum creatinine post surgery (*P*<0.0001), lower urine output on Day 1 (*P* = 0.0286), and greater need for dialysis (*P* = 0.0419). Figure 1 shows the changes of serum creatinine concentrations after liver transplantation for patients who developed AKI and those who did not. During the first 6 hours after transplantation, serum creatinine did not differ significantly between the two groups. Significant differences between groups were seen by 24 hours after surgery and were maintained until 7 days after surgery.

**Table 1:** Descriptive statistics of patient characteristics.

Parameter	AKI	No AKI	*P*
*N*	35	28	–
Age, yr	49.0±11.2	42.4±13.5	0.0475^a^
Male, %	66	70	0.6870^b^
BMI	26.8±6.3	24.0±4.2	0.0735^a^
Surgery time, h	7.1±1.4	6.9±1.3	0.6339^a^
Creatinine change, %	245.7±98.7	13.9±13.6	<0.0001^a^
Urine output (day 1), ml/24 h	1699.8±1178.2	2687.6±1735.6	0.0286^a^
Duration of AKI, d	3.3±2.0	–	–
Hospital stay, d	19.3±18.5	14.0±5.6	0.1359^a^
Dialysis, %	20	4	0.0419^b^

Means ± standard deviation (SD) are reported for continuous measures, percentages are reported for categorical variables.

a Welch modified two-sample *t* test.

b Fisher exact test.

### Associations of Biomarkers with Patient Characteristics

Netrin-1 level, but not sema 3A and NGAL levels, was positively associated with patient age at 2 and 6 hours post-surgery (Table 2). Netrin, sema3A and NGAL levels were not significantly associated with any post-surgery outcome at baseline or at 2, 6, or 24 h following liver transplant (Table 2).

**Table 2:** Spearman correlation coefficients of netrin with clinical characteristics.

	Age	Percent Changein Serum Creatinine	Surgery Time	Hospital Length of Stay	Days AKI
Baseline	0.20	0.10	0.01	−0.08	0.19
2 h	0.30^a^	0.15	−0.04	0.04	0.21
6 h	0.32^a^	0.08	0.11	0.02	0.09
24 h	0.08	0.03	0.03	−0.15	0.12

a *P*≤0.05.

### Urinary Netrin-1 and Sema3A Levels Predict AKI after Liver Transplantation

**Figure 2:**
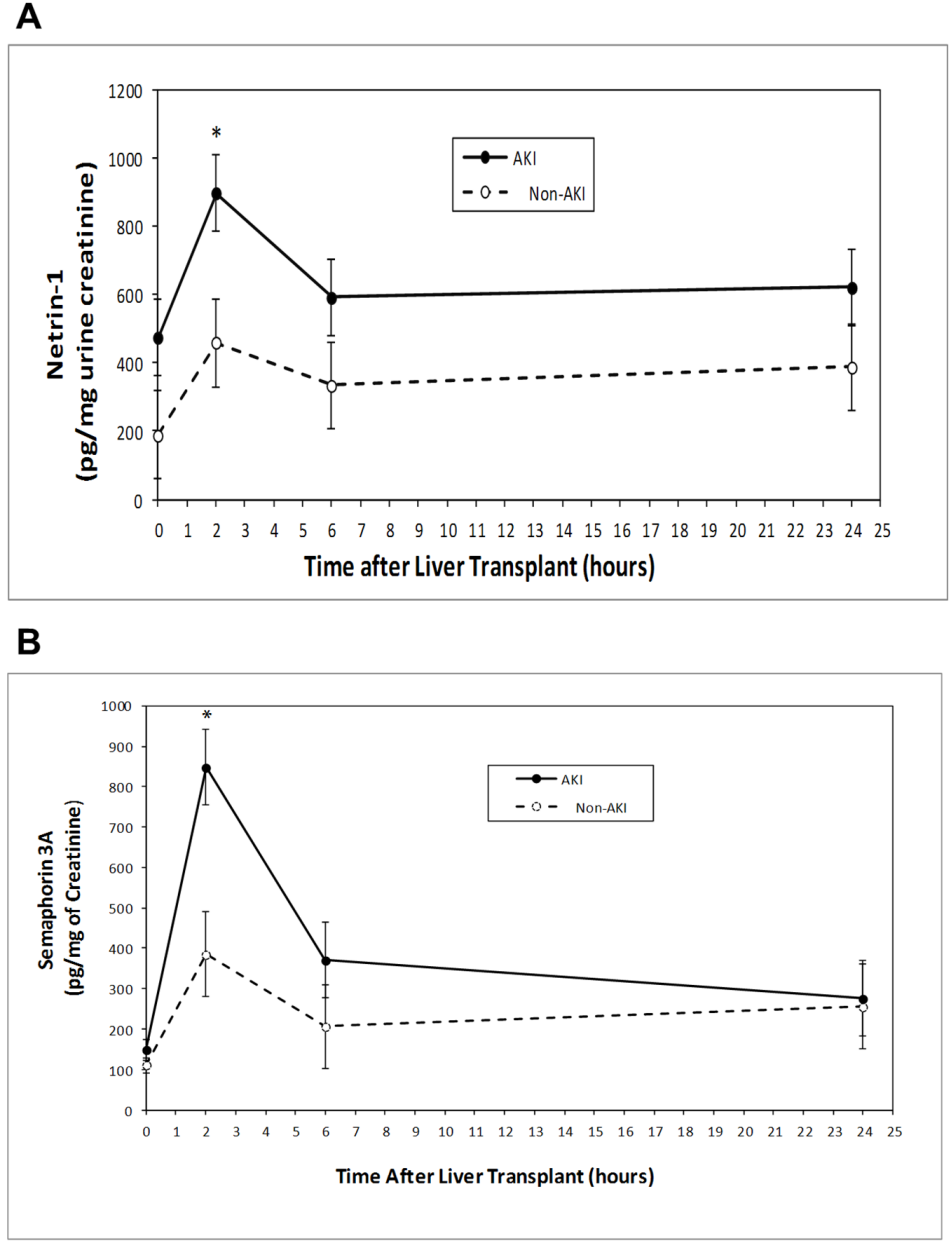
Changes in urinary netrin-1 (A) and sema3A (B) concentrations at various time points after liver transplant surgery in non-AKI and AKI patients. Error bars are LS mean ± SEM. *P = <0.001 for differences between groups (non-AKI and AKI) by repeated-measures ANOVA.

The currently used, but late, diagnostic biomarker for AKI, serum creatinine, begins to rise significantly at 24 hr after liver transplant surgery and remains elevated for the remaining study period in patients categorized as AKI (Figure 1). In contrast, urinary netrin-1 and sema3A levels increased significantly (*P* = 0.0106 and *P* = 0.0012, respectively) and peaked at 2 hours following liver transplant surgery in patients who developed AKI and were no longer significantly elevated at 6 hours after surgery (Figure 2 A and B). Patients who did not develop AKI experienced a much smaller increase in concentration of these biomarker after surgery, which did not differ significantly from baseline. However, the netrin-1 level was relatively higher in the AKI group as compared to the non-AKI group at baseline but this did not reach statistical significance.

**Figure 3:**
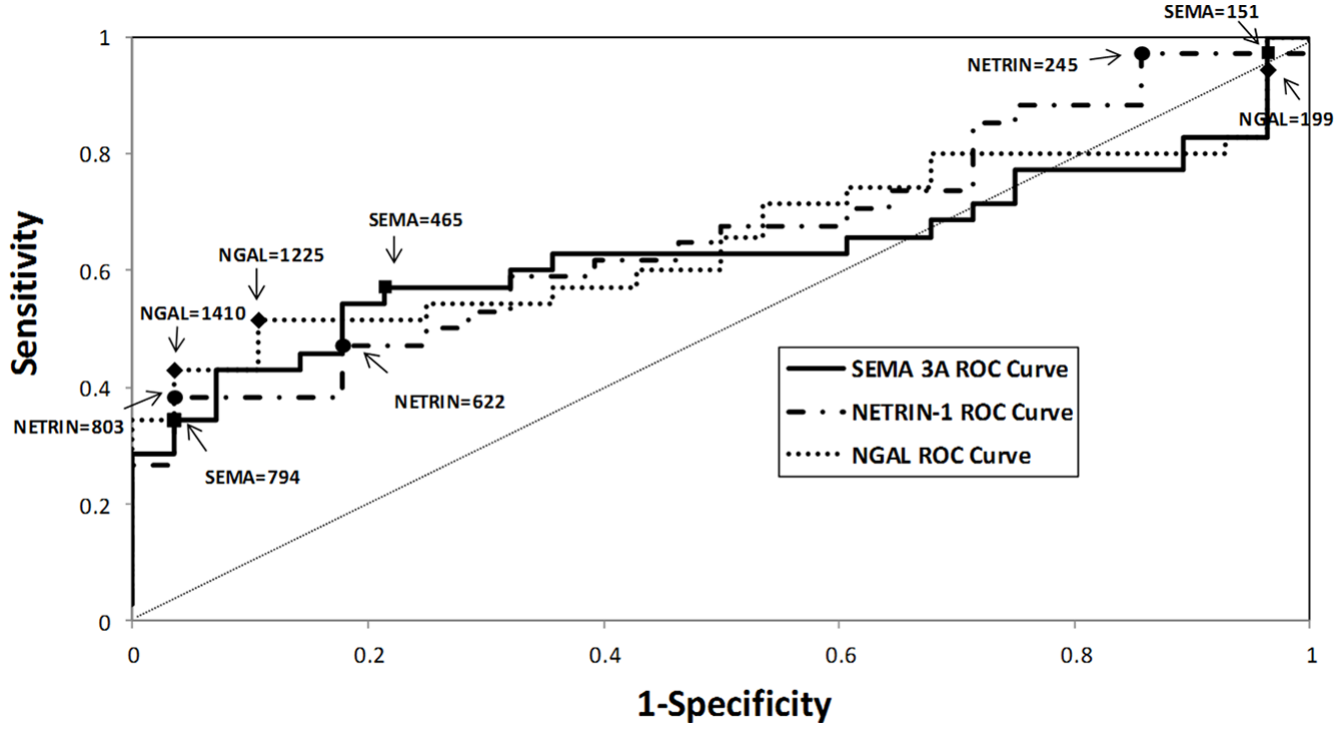
ROC curve analysis for urinary semaphorin 3A, Netrin-1, and NGAL at 2 hours after liver transplantation. The values are urinary concentrations at 2 hours after liver transplant, which correspond to 95% sensitivity, optimal sensitivity and specificity, and 95% specificity, respectively, for each biomarker.

Conventional ROC curves for AKI *versus* no AKI were generated for urinary netrin-1, sema3A and NGAL at 2, 6, and 24 hours after surgery. The AUCs of the three ROC curves for netrin-1 were 0.658 (*P* = 0.0123), 0.570 (*P* = 0.3342), and 0.594 (*P* = 0.1919), respectively. The AUCs of the three ROC curves for sema3A were 0.631 (*P* = 0.0680), 0.560 (*P* = 0.4057), and 0.523 (*P* = 0.7528) respectively. The AUCs of the three ROC curves were 0.651 (*P* = 0.0306), 0.605 (*P* = 0.1369), and 0.603 (*P* = 0.1563), respectively. Thus, the time point for optimal urinary concentration for all three biomarkers was at 2 hours after surgery. Figure 3 displays the unadjusted ROC curve for the three biomarkers at 2 hours after liver transplantation. The sensitivities and specificities for the three biomarkers at optimal concentrations obtained at the 2-hour time point with different combination analyses are listed in Table 3, corresponding to 95% sensitivity, optimal sensitivity and specificity and 95% specificity. All three biomarkers performed equally well when analysed individually. The simultaneous occurrence of levels of 2 urine biomarkers above a designated threshold did not improve the AUC for the prediction of AKI (e.g., when biomarkers were taken in pairs, i.e., 2 by 2), while the AUC improved significantly only when at least 1 of the 3 biomarker urine levels was above threshold.

**Table 3:** Test characteristics for various combinations of biomarkers at 2 hr post-surgery.

Biomarker or Combination	AUC	Sensitivity	Specificity	Positive Predictive Value	Negative Predictive Value
SEMA at 464.5	0.631	0.57	0.79	0.77	0.59
NETRIN at 621.9	0.658	0.47	0.82	0.76	0.56
NGAL at 1225.3	0.651	0.51	0.89	0.86	0.60
SEMA + NETRIN	0.582	0.34	0.82	0.71	0.50
SEMA + NGAL	0.707	0.49	0.93	0.89	0.59
NETRIN + NGAL	0.625	0.29	0.96	0.91	0.52
**At Least 1 Positive**	**0.732**	**0.71**	**0.75**	**0.78**	**0.68**
At Least 2 Positive	0.664	0.54	0.79	0.76	0.58
All 3 Positive	0.625	0.29	0.96	0.91	0.52

**Figure 4:**
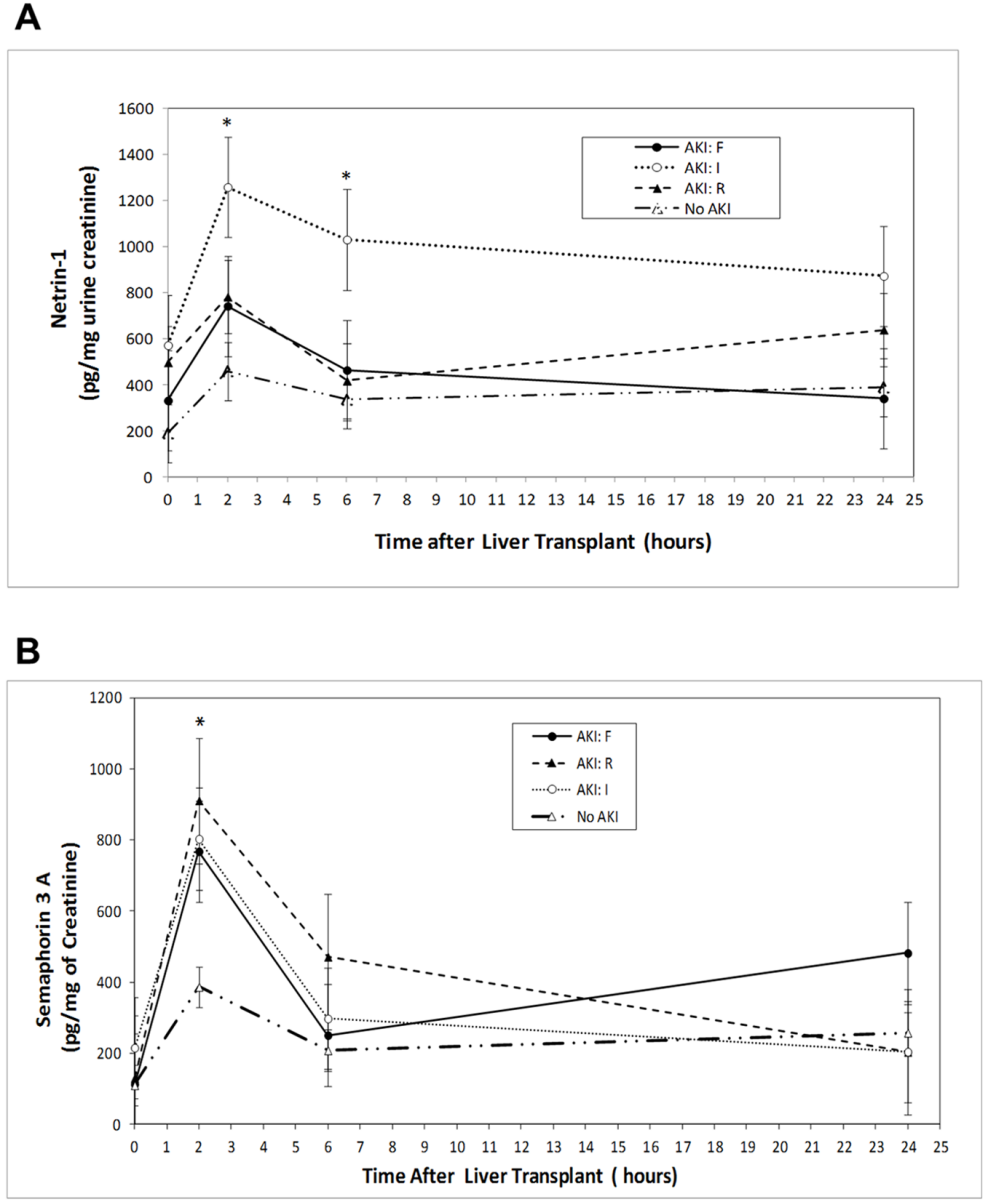
Changes in urinary netrin-1 (A) and sema3A (B) concentrations at various time points after liver transplant surgery in non-AKI and AKI patients, stratified by RIFLE categories. *p≤0.0070 for differences between groups (non-AKI and each of the RIFLE categories) by repeated-measures ANOVA.

Among the 35 subjects who developed AKI, 17 (27%) were classified as being in the risk (R) category, 9 (14%) in the injury (I) category, and 9 (14%) in the failure (F) category, on the basis of RIFLE criteria. Analysis of netrin-1 concentrations by RIFLE classification revealed that the injury group differed from no AKI at 2 hours and 6 hours after OLT (all *P*<0.0070; Figure 3). No other significant differences were found among the RIFLE groups. However, analysis of sema3A concentrations by RIFLE classification revealed that the risk group differed significantly from no AKI at 2 hours (*P* = 0.0026; Figure 4).

Univariable logistic regression identified that age (*P* = 0.0471), urine output on Day 1 (*P* = 0.0265), and higher netrin-1 concentrations at 2 hours (*P* = 0.0369) are significantly associated with higher odds of AKI. A stepwise logistic regression analysis was used to determine the most parsimonious model, given a set of potential variables for predicting AKI. Potential variables for this model included age, sex, BMI, urine output on Day 1, surgery time, hospital length of stay and netrin-1 level at the selected optimal time point (i.e., 2 hours after liver transplant surgery). The final model revealed that BMI and biomarker concentrations at 2 hours after surgery were the only significant independent predictors of AKI in our cohort. The estimated odds ratio for every 1- pg/mg of urinary creatinine increase of netrin-1 at 2 hours after surgery was 1.003 (95% confidence interval: 1.000 to 1.006; *P* = 0.0364). The estimated odds ratio for every 1 unit increase in BMI was 1.198 (95% CI: 1.030 to 1.394; *P* = 0.0194). The estimated odds ratio for every 1- pg/mg of urinary creatinine increase of sema3A at 2 hours after surgery was 1.004 (95% confidence interval: 1.001 to 1.007; *P* = 0.0166). The estimated odds ratio for every 1 unit increase in BMI was 1.238 (95% CI: 1.049 to 1.460; *P* = 0.0113). The estimated odds ratio for every 1 ng/mg of urinary creatinine increase of NGAL at 2 hours after surgery was 1.001 (95% confidence interval: 1.000 to 1.002; *P* = 0.0069). The estimated odds ratio for every 1 unit increase in BMI was 1.225 (95% CI: 1.043 to 1.439; *P* = 0.0135).

## Discussion

In this study, we validated two new biomarkers that have never been studied in liver transplant patients for the diagnosis of AKI. Based on our earlier studies, we chose to examine biomarkers at very early time points i.e., 2, 6 and 24 hr, after transplantation. Our results show that both netrin-1 and sema3A were able to predict the development of AKI similar to known established biomarkers such as NGAL. Our analysis, based on RIFLE classification, shows that the netrin-1 level is much higher in the injury group whereas the sema3A levels is elevated in the risk, injury and failure groups. Interestingly, different combinations of biomarkers did not improve the sensitivity and specificity, as compared to the individual biomarkers, except when at least one marker was considered positive for the prediction of AKI.

Post-operative serum creatinine level begins to rise by 24 hr and remain significantly elevated until day 7 in the AKI group as compared to the non-AKI group. Interestingly, however, we found that the pre-operative serum creatinine level tends to be lower in the AKI group than in the non-AKI group although the difference did not reach statistical significance. The baseline creatinine difference before surgery suggests that AKI patients did not harbor any renal dysfunction prior to transplantation, which was further supported by the similar low basal levels of NGAL and sema3A in both groups. It is interesting to note that pre-operative urine netrin-1 levels tended to be higher in the AKI group as compared to the non-AKI group. However, the reason behind this elevation is unknown. Using serum creatinine elevation to define AKI, we found statistically significant elevation in post-operative urine netrin-1, sema3A and NGAL levels at 2 hr, which then decreases in the subsequent time points. These results are remarkably similar to earlier studies in cardiac bypass surgery patients for netrin-1 and sema3A [9], [12] and in liver transplant patients for NGAL [22]. ROC curve analysis showed that urine netrin-1 levels had a slightly better diagnostic performance as compared to sema3A and NGAL levels, however, it did not reach statistical significance. A number of studies recently evaluated NGAL, but not netrin-1 and sema3A, as a biomarker of AKI in the OLT population. In 2009, Niemann et al. evaluated plasma NGAL levels in 59 patients undergoing OLT and found that elevation in NGAL levels measured at two hours after reperfusion was predictive of AKI [22]. Among those patients with pre-operative serum creatinine levels of <1.5 mg/dL, the plasma NGAL level at two hours post-perfusion was associated with the subsequent development of AKI. The following year, Portal et al. evaluated both urinary and serum NGAL levels in 95 patients undergoing OLT and found that post-operative serum NGAL (but not urinary NGAL) was a predictor of severe AKI using multiple logistic regression analysis [23]. In 2011, Wagener et al. examined urinary NGAL/creatinine ratios in 92 patients undergoing OLT [24]. Elevations in urinary NGAL/creatinine ratios were detected three hours after reperfusion and were more pronounced in patients who developed AKI. Urinary NGAL/creatinine ratios were evaluated again in a recent study by Jeong et al. [25]. Elevated urinary NGAL/creatinine ratios were seen two hours after reperfusion in 11 patients who developed AKI after liver transplantation from a living, related donors and peak elevation was preceded by a rise in serum creatinine in these patients by 19 hours. However, NGAL itself has a varying degree of performance in different settings. Therefore, there is a need to study other biomarkers to determine their relative performance and to see whether a combination of biomarkers can be used for the diagnosis of AKI.

It is interesting to note that the baseline level of netrin-1 in the AKI group was relatively higher than the non-AKI group. The reason for this difference is not clear. However it could be due to the presence of higher levels of renal stressors including circulating factors, hemodymic changes or other unknown factors that may have influence netrin-1 expression in this group. Further studies are required to determine the significance of this observation. Previous studies suggest that netrin-1 is also induced and excreted after kidney transplantation [10] as well other forms of AKI. The induction of netrin-1 was localized in the proximal tubular epithelial cells in response to injury [26], whereas sema3A expression is localized in distal and collecting tubules [12], which is similar to NGAL expression. Based on previous studies, urine netrin-1 most likely comes from proximal tubular epithelium of the kidney. The contribution of serum netrin-1 to urine netrin-1 is unlikely due to its large size (72 KDa). Similarly, the mature form of sema3A has a molecular weight of 95 KDa, which may not be filtered under normal conditions. However, it is also possible that due to a change in hemodynamics, netrin-1 and sema3A may be filtered from the blood and may contribute to urine netrin-1 and sema3A levels in the disease state. This needs to be clarified by future studies. Animal studies show that netrin-1 plays a protective role in epithelial cells [18], [19] whereas sema3A may have a pathogenic role [17] in kidney disease. The molecular mechanism of sema3A induction is less clear. However, netrin-1 expression was shown to be regulated at the translational level [27]. Recently, two biomarkers, insulin like growth factor binding protein-1 (IGFBP-1) and tissue inhibitor of metalloprotease-2 (TIMP-2), were shown to have high specificity and sensitivity for detecting AKI in two different studies, as compared to other previously described biomarkers such as NGAL, KIM-1, IL-18, NAG and liver fatty acid binding protein-1 [28], [29]. However, the performance of IGFBP-1 and TIMP-2 was not validated in OLT patients. Moreover, no studies have compared these biomarkers with netrin-1 and sema3A. Further studies are needed to determine whether netrin-1 and sema3A, either alone or in combination with IGFBP-1 and TIMP-2, can perform better for early diagnosis of AKI in different settings.

Our study has several noteworthy strengths. First, the incidence of AKI in our OLT patient population is 56%, which is similar to what was reported in the literature [5], [22], [25], [30]. Second, netrin-1 and sema3A were examined in an OLT patient population for the first time. Third, netrin-1 performed better than NGAL and sema3A performed similarly to NGAL. Fourth, by examining earlier time points, we found peaks levels of biomarkers that would have been overlooked in previous studies [8]. Our study also has limitations. It is a single-center study. Second, the AUC achieved with all three biomarkers is moderate. Third, the absence of an evaluation of renal function (e.g., serum creatinine, eGFR) months after OLT makes it difficult to know the real clinical impact of the early netrin-1 and sema3a urine levels on the long-term outcome of renal function in OLT recipients.

## Conclusion

The present study establishes the utility of measuring the newly discovered biomarkers netrin-1 and sema3A as biomarkers of AKI in liver transplantation patients. This work should prompt further research into the use of these biomarkers along with urine NGAL to detect AKI prior to the randomization of therapeutic strategies in clinical studies. Given the prevalence of AKI associated with OLT and its associated morbidity and mortality, therapeutic studies based on the diagnosis of AKI using a panel of biomarkers including netrin-1, sema3A, IGFBP-1 and TIMP-2 [28] may be possible in the future.
